# Facility Delivery, Postnatal Care and Neonatal Deaths in India: Nationally-Representative Case-Control Studies

**DOI:** 10.1371/journal.pone.0140448

**Published:** 2015-10-19

**Authors:** Shaza A. Fadel, Usha Ram, Shaun K. Morris, Rehana Begum, Anita Shet, Raju Jotkar, Prabhat Jha

**Affiliations:** 1 Centre for Global Health Research, St Michael’s Hospital, and Dalla Lana School of Public Health, University of Toronto, Toronto, Canada; 2 Department of Public Health and Mortality Studies, International Institute for Population Sciences, Mumbai, India; 3 Division of Infectious Diseases and Centre for Global Child Health, Hospital for Sick Children, Toronto, Canada; 4 Department of Pediatrics, University of Toronto, Toronto, Canada; 5 Department of Pediatrics, St. John’s Medical College Hospital, Bangalore, India; 6 National Health Mission, Government of Maharashtra, Mumbai, India; Centre Hospitalier Universitaire Vaudois, FRANCE

## Abstract

**Objective:**

Clinical studies demonstrate the efficacy of interventions to reduce neonatal deaths, but there are fewer studies of their real-life effectiveness. In India, women often seek facility delivery after complications arise, rather than to avoid complications. Our objective was to quantify the association of facility delivery and postnatal checkups with neonatal mortality while examining the “reverse causality” in which the mothers deliver at a health facility due to adverse perinatal events.

**Methods:**

We conducted nationally representative case-control studies of about 300,000 live births and 4,000 neonatal deaths to examine the effect of, place of delivery and postnatal checkup on neonatal mortality. We compared neonatal deaths to all live births and to a subset of live births reporting excessive bleeding or obstructed labour that were more comparable to cases in seeking care.

**Findings:**

In the larger study of 2004–8 births, facility delivery without postnatal checkup was associated with an increased odds of neonatal death (Odds ratio = 2.5; 99% CI 2.2–2.9), especially for early versus late neonatal deaths. However, use of more comparable controls showed marked attenuation (Odds ratio = 0.5; 0.4–0.5). Facility delivery with postnatal checkup was associated with reduced odds of neonatal death. Excess risks were attenuated in the earlier study of 2001–4 births.

**Conclusion:**

The combined effect of facility deliveries with postnatal checks ups is substantially higher than just facility delivery alone. Evaluation of the real-life effectiveness of interventions to reduce child and maternal deaths need to consider reverse causality. If these associations are causal, facility delivery with postnatal check up could avoid about 1/3 of all neonatal deaths in India (~100,000/year).

## Introduction

India’s under—five child mortality rate has fallen by more than half since 1990 (from 115 to 49 per 1,000 live births in 2013) [1, 2]. An increasing proportion of under—five deaths occur during the neonatal period, accounting for nearly three-fifths of under—five deaths in 2013 compared to two-fifths in 1990 [1, 2]. At a rate of 28 deaths per 1,000 live births, India has the highest number of annual neonatal deaths globally (~0.8 million) [2, 3]. About four-fifths of neonatal deaths occur within the first week, and about four-fifths of neonatal deaths are due to three causes: prematurity and low-birth weight, infections, or birth asphyxia and birth trauma [1, 4].

India's *Janani Suraksha Yojana* (JSY) cash transfers have raised the prevalence of institutional deliveries, but evaluations show that neonatal mortality has not declined in unison [5]. Moreover, low quality emergency obstetric referral services are associated with maternal and perinatal deaths [5–7]. Health interventions are well known to improve health outcomes for mother-infant pairs in clinical studies and in randomized trials [8, 9]. These interventions can be separated into six packages along a continuum of care that encompass: preconception nutrition care, antenatal care, care during labour and childbirth, immediate newborn care, care for the healthy neonate, and specific interventions to care for small and ill neonates [8]. It has been difficult to quantify the population-level effectiveness of each of these interventions because their use is highly correlated and can be complicated by “reverse causality” when treatment is sought only after serious illness has ensued [10]. In particular, women of lower socioeconomic status (SES) tend to use these interventions less than higher SES groups, and practices and treatment seeking behaviours among lower SES women [11–13] complicate assessment of the real-life effectiveness of interventions at the population-level. Delays in receiving care from trained medical personnel [7, 14] have been observed in India and in Africa [15–17] and involve one or more of: failure to recognize severity of symptoms, seeking treatment first from unqualified practitioners, inaccessibility to emergency obstetric care, delays in referral between institutions, and reduced quality of care [6, 7, 9].

In this report, we use nationally-representative household survey data to quantify the association of facility delivery and postnatal checkups with neonatal mortality. We specifically examine the “reverse causality” in which the mothers deliver at a health facility due to adverse perinatal events, and not to avoid them [14–17].

## Materials and Methods

### Data and Study Design

We conducted case control studies using live births and neonatal deaths reported in the District Level Household Survey-3 (DLHS-3), a national household survey conducted in 2007–8 using a multi-stage stratified sampling in all Indian districts (small administrative areas each with about 2 million people) [18]. A total of 643,944 ever-married women aged 15–49 years were surveyed from 601 districts, of which 216,891 had one or more live births within the three years prior to the survey (2004–8). The DLHS surveys interviewed women directly and therefore live births and cases of death exclude children whose mother had died prior. Exclusions were 896 births with missing information on neonatal survival, 4,003 twins or triplets, and 36 births where mother reported two singleton births in the same year. We also excluded 936 births who died on day 0 because they did not have an equal opportunity to receive a postnatal checkup within 24 hours (S1 Table). The final analysis was of 211,020 live births among whom 2,530 neonatal deaths occurred. Similar approaches and exclusions were followed for DLHS-2, which is also district representative [19]. DLHS-2 interviewed mothers about births from 2001–4, and after similar exclusions (S1 Fig), the final analyses was of 92,453 live births among whom 1,573 neonatal deaths occurred.

Cases were defined as deaths between 1–28 days among most recent live births. The main analyses use two types of controls: (i) all live births alive at day 28 (hereafter called “all controls”); and (ii) a subset of live births where mothers reported seeking treatment for excessive bleeding during pregnancy or obstructed labour. Excessive bleeding, obstructed and prolonged labour, and maternal infection are the most common reasons mothers are referred to health facilities during pregnancy and for delivery in the South Asian context [7]. The latter controls were chosen to examine possible reverse causality and hereafter called “more comparable controls.”

### Analysis

#### Variable and model selection

We developed a conceptual model to select variables capturing community, social and clinical care features, and we tested similar variables to minimize collinearity. Facility deliveries included those that would have occurred in government, NGO or non-profit and private facilities. The postnatal checkup variable was derived from questions on a) whether the newborn received a checkup within 24 hours, b) the number of checkups the newborn received within ten days, and c) the location of the first newborn checkup. We did not find a major difference between postnatal checks ups performed at health facilities described above and those delivered at home by a doctor or auxiliary nurse midwife (a village-level health care worker focused on maternal and child health) and thus combined them. Antenatal care interventions—maternal tetanus toxoid, antenatal care visits, and iron folic acid consumption—were highly correlated (data not shown). Thus the subsequent analyses used maternal tetanus toxoid to capture exposure to all three interventions; results were similar using the other two variables. We could not measure the effect of breastfeeding in our models. There were no questions on breastfeeding for any child who had died in DLHS-2. In DLHS-3, responses to the question on initiation of breastfeeding were missing when mothers reported that the child did not survive to receive a postnatal check up within 24 hours of birth, even if the child died after 24 hours.

Logistic regression models were built for the primary outcome of neonatal death, with further stratification of early (1–6 days) and late (7–28 days) neonatal deaths. For the DLHS-3 analysis, we added an interaction term between the place of delivery and postnatal checkup because of the strong association between the two variables and because both interventions would likely be delivered within the same time frame. The reference group for comparisons related to the interaction was live births whose mothers reported having unattended home delivery (home delivery in the absence of a skilled birth attendant) and no postnatal checkup. Tests for significance of confounding and interaction used Kleinbaum’s procedure [20]. Full models with an interaction term yielded better measures of goodness of fit and model adequacy compared to analyses stratified by place of delivery. Significance of the odds ratios (OR) for neonatal death were measured by the fixed effects (inverse variance) method [21]. We treated each model as a separate study and used the test for heterogeneity to assess differences across models [21]. We defined facility deliveries similarly n DLHS-2 and DLHS-3 analyses. In DLHS-2, postnatal check ups were only measured if they occurred as home-based visits. Thus, for DLHS-2 analyses no interaction term was added for place of delivery and postnatal check up.

Population level prevalence of the coverage of effective interventions were calculated from the DLHS-3 for the nine poorer states (in order of absolute neonatal death totals: Uttar Pradesh, Bihar, Madhya Pradesh, Rajasthan, Orissa, Assam, Jharkhand, Chhattisgarh, Uttarakhand) and the remaining richer states. We estimated neonatal deaths in India for 2014 using methods previously described for 2013 [2]. State-specific neonatal deaths were calculated and then summed to generate national-totals. Proportions and 99% confidence intervals accounted for survey-design using DLHS sample weights. The Population Prevented Fraction (PPF) was calculated using prevalence of exposures among the live births occurring in the last three years prior to the survey multiplied by 1-adjusted relative risk ratio. We used Stata v12.1 (StataCorp, LP, College Station, TX) for the analyses.

## Results

Cases and all controls in the DLHS-3, respectively, had similar proportions of unattended home delivery with or without postnatal checkup (51% vs. 48%) and of facility delivery (44% vs. 47%). There were sharp differences between cases and controls in proportions of postnatal checkups (35% vs. 51%), especially among facility deliveries (19% vs. 37%). The other correlates of neonatal death were well described [22]: cases had lower maternal tetanus toxoid coverage, were more likely males, were more likely to have mothers at high risk ages of 12–19 years or 35–49 years, illiterate mothers, and were from the poorer states of India (Table 1).

**Table 1:** Prevalence of exposures and adjusted odds ratios among singleton live births who died or survived the neonatal period by two different controls, India 2004–2008.

	Cases: Day 1–28 deaths (n = 2,530)	All Controls (n = 208,490)		Controls reporting excessive bleeding or obstructed labour (n = 11,205)	
	Number/ Percent^c^	Number/ Percent^c^	Adjusted OR (99% CI)	Number/ Percent^c^	Adjusted OR (99% CI)
**Unattended home delivery**	**1,361/51.2%**	**106,910/47.8%**		**1,099/8.6%**	
and no postnatal checkup	1,143/42.6%	86,944/38.5%	Ref.	793/6.1%	Ref.
and postnatal checkup	150/6.0%	15,966/7.5%	0.78 (0.59, 1.03)	257/2.1%	0.47 (0.33, 0.66)
and indeterminate postnatal checkup^a^	68/2.6%	4,000/1.8%		49/0.4%	
**Facility delivery**	**1,039/43.6%**	**89,560/46.5%**		**9,821/89.0%**	
and no postnatal checkup	470/19.1%	15,654/7.6%	2.54 (2.21, 2.92)	900/7.4%	0.46 (0.39, 0.54)
and postnatal checkup	428/18.6%	70,185/37.0%	0.59 (0.51, 0.69)	8,306/76.2%	0.05 (0.04, 0.06)
and indeterminate postnatal checkup^a^	141/5.9%	3,721/1.9%		615/5.4%	
**Home delivery with skilled attendant**	**115/4.7%**	**11,797/5.7%**		**283/2.3%**	
and no postnatal checkup	84/3.3%	6,290/3.0%	1.09 (0.79, 1.51)	113/0.9%	0.65 (0.43, 0.98)
and postnatal checkup	23/1.0%	5,023/2.5%	0.41 (0.24, 0.69)	142/1.2%	0.15 (0.09, 0.24)
and indeterminate postnatal checkup^a^	8/0.4%	484/0.2%		28/0.2%	
missing delivery or postnatal checkup	15	223		2	
No Maternal tetanus toxoid	885/33.3%	59,829/26.7%	Ref.	1,171/9.5%	Ref.
> 1 Maternal tetanus toxoid	1,642/66.6%	148,561/73.2%	0.95 (0.81, 1.10)	10,032/90.5%	0.66 (0.54, 0.80)
Missing	3			2	
Female	1,087/43.0%	96,761/46.4%	Ref.	5,217/46.6%	Ref.
Male	1,439/56.8%	111,720/53.6%	1.15 (1.04, 1.28)	5,988/53.4%	1.24 (1.07, 1.43)
missing	4			0	
Low risk (20–34 y/o)	1,880/74.6%	171,020/82.7%	Ref.	9,304/84.0%	Ref.
High risk (12–19 y/o, 35–49 y/o)	650/25.4%	37,470/17.4%	1.51 (1.35, 1.69)	1,901/16.0%	1.25 (1.03, 1.46)
Mother attended > 5 years of school	946/39.7%	99,041/50.7%	Ref.	7,744/72.0%	Ref.
Mother attended < 5 years of schooling	1,584/60.3%	109,438/49.4%	1.30 (1.15, 1.47)	3,459/28.0%	1.55 (1.33, 1.81)
missing	0			2	
Richer states	756/33%	85,780/44.4%	Ref.	5,696/52.7%	Ref.
Poorer states^b^	1,774/67.0%	122,710/55.6%	1.30 (1.12, 1.50)	5,510/47.3%	1.05 (0.86, 1.28)

^a^ Indeterminate postnatal checkups refers to singleton live births for which mothers reported a newborn postnatal checkup within ten days but the location was unspecified.

^b^ Poorer states are EAGA states: Empowered Action Group and Assam which encompass Uttar Pradesh, Bihar, Madhya Pradesh, Rajasthan, Orissa, Assam, Jharkhand, Chhattisgarh, Uttarakhand. The remaining states and union territories are classified as richer states.

^c^ The numbers presented in table are unweighted and the proportions are weighted.

The prevalence of home delivery with skilled birth attendant (with or without postnatal checkup) was less than 6% in cases or controls. Nevertheless, in comparison with unattended deliveries and no postnatal check ups, the presence of a skilled attendant during a home delivery did not reduce the odds of neonatal death. By contrast, postnatal checkups after home deliveries with a skilled birth attendant were associated with reduced odds of neonatal deaths (OR = 0.41) (Table 1).

**Fig 1:**
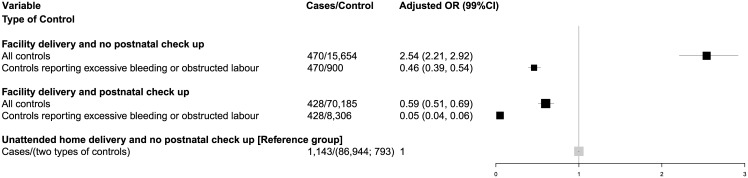
Adjusted odds ratios for neonatal death for facility deliveries by different controls, India 2004–08.

**Fig 2:**
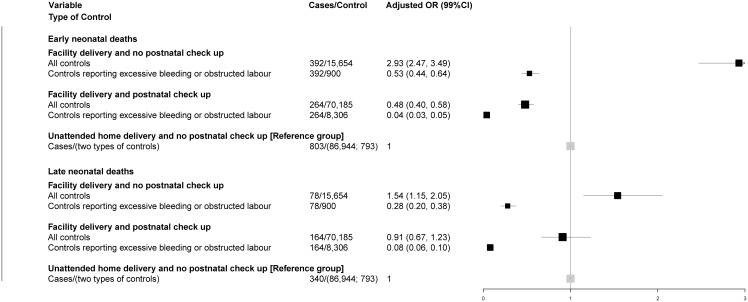
Adjusted odds ratios for early neonatal deaths and late neonatal deaths for facility deliveries by different controls, India 2004–08.

The remaining analyses focus on the OR of neonatal death among facility deliveries as compared to the reference group of those with unattended home delivery without a postnatal checkup. Adjusting for maternal age and education, tetanus toxoid during pregnancy, sex of child, and residency in poor states, elevated the odds of a neonatal death to OR = 2.5 for facility delivery without postnatal checkup (Fig 1). The adjusted odds of early neonatal death (OR = 2.9) was elevated higher than late neonatal death (OR = 1.5; Fig 2). By contrast, facility delivery with postnatal checkup reduced the prevalent odds for all neonatal deaths (OR = 0.6) and early neonatal deaths (OR = 0.5), but not significantly associated (OR = 0.9) with late neonatal deaths.

The elevated odds of neonatal death for facility delivery without postnatal checkup was unexpected, and we explored the possibility that cases were likely to seek facility delivery as a result of delayed care, rather than a planned facility delivery, while few of the controls would have this bias. We examined the odds of neonatal death to more comparable controls, specifically live births reporting excessive bleeding or obstructed labour. More comparable controls were far more likely to report their birth occurred in a health facility than were all live births (OR 10.42, 99% CI 9.65, 11.24).

Use of these more comparable controls substantially attenuated the elevated, adjusted odds of neonatal deaths with facility delivery without postnatal checkup (OR = 0.5; Fig 1). Early and late neonatal deaths were similarly attenuated (Fig 2). The more comparable controls resulted in facility delivery with postnatal checkup associated with a greater reduction in odds of neonatal death (OR = 0.05), and similarly for early and late neonatal deaths.

**Fig 3:**
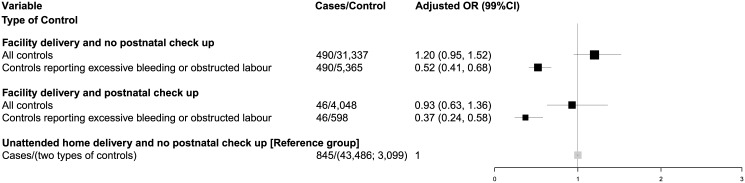
Adjusted odds ratios for neonatal death for facility deliveries by different controls, India 2001–04. Note: Postnatal check ups were measured as home visits within two weeks after delivery. Postnatal check ups occurring at health facilities were not measured. There is no significant interaction effect between home postnatal visits alone with place of delivery.

Analyses of cases and controls from the DLHS-2 surveying births from 2001 to 2004 were broadly similar (Fig 3; S1 Table). The odds of neonatal death for facility delivery, with or without postnatal checkup, were attenuated when using more comparable controls. There was a modest decrease from the DLHS-2 to DLHS-3 time periods among all controls in any unattended home delivery (52% to 48%) and a modest increase in facility delivery (41% to 47%; Table 1 and S1 Table). These changes were similar to those reported among neonatal deaths. Between the two survey periods and among more comparable controls, dramatic decreases were seen in unattended home delivery (33% to 9%), and dramatic increases were seen in facility delivery (60% to 89%). DLHS-2 defined postnatal checkups as home visits only, so the trends are undetermined.

We next calculated the number of neonatal deaths that could be avoided with facility delivery and postnatal checkups, taking into account reverse causality. We combined DLHS-3 estimates of coverage and 2014 estimates of neonatal deaths, with the 99% upper and lower confidence intervals of relative risks calculated from the multivariable model in Table 1. Facility delivery combined with postnatal checkups would have avoided about 33–34% of neonatal deaths, while facility delivery without postnatal checkup would avoid less than 3% of neonatal deaths. In poorer states, the currently low prevalence of postnatal checkups after facility deliveries yields only 20–21% avoidable neonatal deaths, which is less than half of the proportion of about 46–47% of avoidable neonatal deaths in the richer states. However, as two thirds of all neonatal deaths occur in the poorer states, the absolute numbers of avoidable deaths are about 100,000 in the poorer and richer group of states (Table 2).

**Table 2:** Prevalence of exposures, population prevented fraction and preventable number of neonatal deaths in 2014 in India, poorer states and richer states.

	India			Poorer states			Richer states		
Intervention	Prevalence (%)^a^	PPF^b^	Preventable number of deaths^c^	Prevalence (%)^a^	PPF^b^	Preventable number of deaths^c^	Prevalence (%)^a^	PPF^b^	Preventable number of deaths^c^
**Facility delivery and no postnatal checkup**	8%	2–3%	20,000–23,000	9%	3–4%	15,000–17,000	6%	2%	5,000–6,000
**Facility delivery and postnatal checkup**	37%	33–34%	245,000–251,000	23%	20–21%	101,000–104,000	55%	46–47%	115,000–118,000

^a^Prevalence of exposures was based on live births during the past three years prior to the survey.

^**b**^ Population Prevented Fraction = Prevalence of exposure x(1-RR) and was calculated for neonatal deaths using adjusted relative risks calculated when Using controls whose mothers reported excessive bleeding and obstructed labour as in the model reported in Table 1. RR for facility delivery and no postnatal check up was 0.64 (99% CI 0.61, 0.67), and the RR for facility delivery and postnatal check up was 0.10 (99% CI 0.09, 0.11).

^c^Calculated using 2014 estimates of neonatal mortality in India. There were approximately 745,00 estimated number of neonatal deaths in India, 496,000 in poorer states and 249,000 in richer states.

## Discussion

Using two nationally representative case-control studies of live births occurring between 2001 and 2008 in India, we demonstrate that basic interventions along the continuum of care are strongly associated with reduced neonatal deaths. In particular, neonatal deaths were notably lower when postnatal checkups were combined health facility delivery, in contrast to facility delivery alone. Our estimates take into account the bias that the sickest children/mothers tend to preferentially deliver in facilities, by restricting controls to those whose mothers reported obstetric complications [10, 23].

Postnatal checkups have been associated with improved neonatal survival specifically when related to reducing cord infections, early identification of illness or management of low birth weight babies [24–26]. Immediate postnatal assessment and neonatal resuscitation techniques on the day of birth are critical for increasing the likelihood of neonatal survival if perinatal birth asphyxia or birth trauma occurs [8, 27, 28]. However, approximately 20% of Indian women who delivered in a health facility in 2008–2009 reported their duration of stay as less than 24 hours, more commonly among women from poorer states [29]. 60% and 53% of women surveyed in Bihar and Uttar Pradesh, the states with the highest neonatal mortality rates in India, were discharged in less than 24 hours [29]. Our results further emphasize the importance of postnatal checkups as part of a facility-based delivery package.

The World Health Organization and United Nations Children’s Fund recommend that postnatal care visits performed by a qualified health worker for all neonates are initiated within 24 hours of birth, day 3, and day 7 after birth [30, 31]. During the DLHS-3 survey period, India’s Integrated Management of Neonatal and Childhood Illness programme for home-based delivery of interventions was in the introductory phase in most states, and JSY has led to increase in the prevalence of institutional deliveries [5, 26]. The Government of India’s strategy is to increase coverage of postnatal checkups by 7.5% annually [32]. However, only home-based postnatal visits for newborns delivered at home are currently being tracked by the National Rural Health Mission [32].

There is little doubt about the clinical efficacy of the interventions delivered during facility delivery and postnatal checkups to improve obstetric and newborn care [33]. However, their real-life effectiveness has been questioned [10, 34, 35]. Various studies in low-income countries (including India) showed that maternal and perinatal mortality does not improve, and indeed can be raised among women who seek care at health facilities [10, 34, 35]. Poor quality of comprehensive emergency obstetric services is usually the explanation for such paradoxical findings [23]. Our study suggests that reverse causality be considered carefully in real-life effectiveness studies, and provides a simple method to select controls to at least partially account for reverse causality. Neonatal (or maternal) deaths differ in many ways from those who have uncomplicated deliveries and normal deliveries. As care seeking is increasing, real-life effectiveness studies need to rely on more than just comparisons of deaths with all live controls.

We might have overestimated the effect of facility delivery and postnatal checkups by using more comparable controls. The prevalence of facility deliveries, postnatal checkups and use of maternal tetanus toxoid and maternal education among the more comparable controls was notably higher than among all controls (Table 1). However, there were smaller differences between more comparable controls and all controls in the sex of the child, maternal age at birth and residency in a poor state. Thus, the use of more comparable controls, while addressing reverse causality, might have introduced other selection biases towards better health seeking behaviour, which would differ between neonatal deaths and the more comparable controls (hence overestimating the reduction in odds of death from facility delivery with or without postnatal checkup). Adjusting for covariates such as maternal education partially took these differences into account. We attempted to quantify the size of better health seeking behaviour among the more comparable controls versus all controls by examining the odds of receiving at least one dose of diphtheria, pertussis, and tetanus (DPT) vaccination, which is typically administered six weeks after birth. After adjusting for maternal age, residency in poorer states, sex of the child, maternal education and receipt of maternal tetanus toxoid, the OR of receiving one dose of DPT vaccine was only 1.09 (99%CI 1.02,1.16) in more comparable controls than in all controls (excluding the more comparable controls). This suggests that there is better health seeking behaviour among the more comparable controls, but may only have a modest impact on the large attenuation observed in the effect of facility deliveries and postnatal checkups on neonatal deaths.

We also examined a larger group of controls whose mothers reported prolonged labour, breech presentation, excessive bleeding or obstructed labour (S3 Table). These intermediate controls showed less attenuation of the odds of neonatal death than our more comparable controls. Thus a plausible dose-response relationship was seen. Furthermore, our effect sizes of up to a 90% reduction in the odds of neonatal death with facility delivery and postnatal checkup are congruent with a systematic review of clinical efficacy of interventions; this found that high coverage of interventions along the continuum of care averts about 80% of neonatal deaths [8]. Community-based postnatal care trials performed in South Asia have also shown 30–60% reductions in neonatal mortality [36, 37].

There are several limitations in our study. Early initiation of breastfeeding is an important strategy to promote neonatal well-being [8, 9]; however, breastfeeding data in the DLHS-3 were not missing at random. While multiple imputations for the missing data did not yield significantly different estimates from excluding the variable from our model, breastfeeding remains potentially an unmeasured confounding variable. Our analysis did not measure the quality of emergency obstetric care and newborn care at facilities or community-based newborn care training, all of which are important distal factors that influence neonatal outcome [6, 8, 26]. Finally, neonatal deaths were slightly underrepresented in the study, as multiple births and newborns whose mothers have died were not included.

## Conclusions

Safe delivery is associated with lower odds of neonatal deaths. By using more comparable controls to deaths, the combined effect of facility deliveries with postnatal checks ups is associated with substantially lower odds of neonatal death than just facility delivery alone. If these associations are causal, about a third of all neonatal deaths (~100,000/year) in India can be avoided by facility delivery combined with postnatal checkup. Practicable methods, such as ours, to evaluate the real-life effectiveness of interventions for the mother and infant at the population level need to consider reverse causality.

## Supporting Information

S1 Fig Inclusion and exclusion of live births from the second and third rounds of the District Level Household Survey. (EPS)

S1 Table Prevalence of place of delivery and postnatal checkups among singleton live births who died on day 0, India. (DOC)

S2 Table Prevalence of exposures and adjusted odd ratios among singleton live births who died or survived the neonatal period by two different controls, India 2001–2004. (DOC)

S3 Table Prevalence of exposures and adjusted odds ratios among singleton live births who died or survived the neonatal period by controls reporting some obstetric complications, India 2004–2008. (DOC)
