# Supplementary material for: Facility Delivery, Postnatal Care and Neonatal Deaths in India: Nationally-Representative Case-Control Studies
Source: PLoS One. 2015 Oct 19;10(10):e0140448. doi: 10.1371/journal.pone.0140448 (PMC4610669; doi:10.1371/journal.pone.0140448)
Supplement: S1 Table — (DOC) [file pone.0140448.s002.doc]

**S1 Table.** **Prevalence of place of delivery and postnatal checkups among singleton live births who died on day 0, India.**

|  | **Cases: Day 0 deaths (n=936), 2004-2008** | | **Cases: Day 0 deaths (n=578), 2001-2004** |  |
| --- | --- | --- | --- | --- |
|  | **Number/Percent** | | **Number/Percent** |  |
| Unattended home delivery |  | |  |  |
| and no postnatal checkup | 438/44.6% | | 304/51.3% |  |
| and postnatal checkup | 17/1.9% | | 10/2.0% |  |
| and indeterminate postnatal checkup^1^ | 2/0.2% | |  |  |
|  |  | |  |  |
| Facility delivery |  | |  |  |
| and no postnatal checkup | 338/37.1% | | 192/35.0% |  |
| and postnatal checkup | 53/6.5% | | 12/1.9% |  |
| and indeterminate postnatal checkup | 20/2.4% | |  |  |
|  |  | |  |  |
| Home delivery with skilled attendant |  | |  |  |
| and no postnatal checkup | 51/5.3% | | 43/6.7% |  |
| and postnatal checkup | 9/1.0% | | 12/1.9% |  |
| and indeterminate postnatal checkup | 1/0.2% | |  |  |
|  |  | |  | |
| Missing | 7 | | 4 | |
|  |  |  | | |
