# Supplementary material for: Facility Delivery, Postnatal Care and Neonatal Deaths in India: Nationally-Representative Case-Control Studies
Source: PLoS One. 2015 Oct 19;10(10):e0140448. doi: 10.1371/journal.pone.0140448 (PMC4610669; doi:10.1371/journal.pone.0140448)
Supplement: S2 Table — (DOC) [file pone.0140448.s003.doc]

**S2 Table. Prevalence of exposures and adjusted odd ratios among singleton live births who died or survived the neonatal period by two different controls, India 2001-2004.**

|  | **Cases: Day 1-28 neonatal deaths (n=1,573)** | **All Controls (n=90,880)** | | **Controls reporting excessive bleeding or obstructed labour (n=10,279)** | |
| --- | --- | --- | --- | --- | --- |
|  | **Number/Percent** | **Number/Percent** | **Adjusted OR (99% CI)** | **Number/Percent** | **Adjusted OR (99% CI)** |
| Unattended home delivery | 910/57.4% | 48,357/51.8% | Ref. | 3,581/33.3% | Ref. |
| Facility delivery | 544 /36.1% | 35,402/41.1% | 1.20 (0.95, 1.52) | 5,964/59.8% | 0.52 (0.41, 0.68) |
| Home delivery with skilled birth attendant | 112/6.3% | 7,043/6.9% | 1.01 (0.69, 1.48) | 725/6.82% | 0.63 (0.43, 0.93) |
| missing | 7 | 78 |  | 9 |  |
|  |  |  |  |  |  |
| No postnatal checkup | 1,431/90.2% | 80,051/86.5% | Ref. | 8,997/85.8% | Ref. |
| Postnatal checkup^a^ | 129/9.0% | 10,763/13.4% | 0.78 (0.54, 1.12) | 1,279/14.2% | 0.71 (0.48, 1.04) |
| missing | 13 | 66 |  | 3 |  |
|  |  |  |  |  |  |
| No Maternal tetanus toxoid | 445/27.0% | 18,078/18.1% | Ref. | 1,431/10.6% | Ref. |
| > 1 Maternal tetanus toxoid | 1,127/73.0% | 72, 788/81.9% | 0.73 (0.62, 0.86) | 8,848/89.4% | 0.53 (0.43, 0.64) |
| missing | 1 | 14 |  | 0 |  |
|  |  |  |  |  |  |
| Female | 693/44.2% | 42,747/47.2% | Ref. | 4,843/47.9% | Ref. |
| Male | 880/55.8% | 48,133/52.8% | 1.12 (0.93, 1.35) | 5,436/52.1% | 1.19 (0.95, 1.49) |
|  |  |  |  |  |  |
| Low risk (20-34 y/0) | 1,434/65.0% | 67,668/72.2% | Ref. | 7,680/71.0% | Ref. |
| High risk (12-19 y/o, 35-49 y/o) | 527/36.3% | 23,212/27.7% | 1.46 (1.20, 1.77) | 2,599/29.0% | 1.37 (1.11, 1.69) |
|  |  |  |  |  |  |
| Mother attended > 5 years of school | 527/32.4% | 41,545/44.1% |  | 5,576/52.2% |  |
| Mother attended < 5 years of school | 1,045/67.5% | 49,274/55.9% | 1.43 (1.20, 1.71) | 4,703/47.7% | 1.40 (1.12, 1.76) |
| missing | 1 | 61 |  | 9 |  |
|  |  |  |  |  |  |
| Richer states | 501/32.9% | 41,018/45.8% | Ref. | 5,400/56.2% | Ref. |
| Poorer states^b^ | 1,072/67.1% | 49,862 /54.2% | 1.55 (1.22, 1.98) | 4,879/43.8% | 1.89 (1.48, 2.40) |
| ^a^ Postnatal checkup was measured as home visits within two weeks after delivery. Postnatal checkups occurring at health facilities were not measured. There is no significant interaction effect between home postnatal visits alone with place of delivery.  ^b^ Poorer states are EAGA states: Empowered Action Group and Assam which encompass Uttar Pradesh, Bihar, Madhya Pradesh, Rajasthan, Orissa, Assam, Jharkhand, Chhattisgarh, Uttarakhand. The remaining states and union territories are classified as richer states. | | | | | |
