# Supplementary material for: Facility Delivery, Postnatal Care and Neonatal Deaths in India: Nationally-Representative Case-Control Studies
Source: PLoS One. 2015 Oct 19;10(10):e0140448. doi: 10.1371/journal.pone.0140448 (PMC4610669; doi:10.1371/journal.pone.0140448)
Supplement: S3 Table — (DOC) [file pone.0140448.s004.doc]

**S3 Table.** **Prevalence of exposures and adjusted odds ratios among singleton live births who died or survived the neonatal period by controls reporting some obstetric complications, India 2004-2008.**

|  | **Cases: Day 1-28 deaths (n=2,530)** | **Controls reporting some obstetric complications^c^ (n=42,231)** | |
| --- | --- | --- | --- |
|  | **Number/Percent^d^** | **Number/Percent^d^** | **Adjusted OR (99% CI)** |
| Unattended home delivery | 1,361/51.2% | 13,257/27.9% |  |
| and no postnatal checkup | 1,143/42.6% | 10,209/21.3% | Ref |
| and postnatal checkup | 150/6.0% | 2,484/5.4% | 0.60 (0.45, 0.80) |
| and indeterminate postnatal checkup^a^ | 68/2.6% | 564/1.2% | 1.18 (0.84, 1.66) |
|  |  |  |  |
| Facility delivery | 1,039/43.6% | 27,255/68.4% |  |
| and no postnatal checkup | 470/19.1% | 3,007/6.9% | 1.60 (1.37, 1.86) |
| and postnatal checkup | 428/18.6% | 22,791/57.9% | 0.22 (0.19, 0.26) |
| and indeterminate postnatal checkup^a^ | 141/5.9% | 1,457/3.6% | 1.08 (0.82, 1.41) |
|  |  |  |  |
| Home delivery with skilled attendant | 115/4.7% | 1,704/3.8% |  |
| and no postnatal checkup | 84/3.3% | 774/1.7% | 1.07 (0.75, 1.53) |
| and postnatal checkup | 23/1.0% | 829/1.9% | 0.29 (0.18, 0.49) |
| and indeterminate postnatal checkup^a^ | 8/0.4% | 101/0.2% | 0.90 (0.31, 2.63) |
|  |  |  |  |
| No Maternal tetanus toxoid | 885/33.3% | 8,510/18.0% | Ref. |
| > 1 Maternal tetanus toxoid | 1,642/66.6% | 33,717/81.9% | 0.94 (0.80, 1.11) |
| missing | 3 | 4 |  |
|  |  |  |  |
| Female | 1,087/43.0% | 19,522/46.3% | Ref. |
| Male | 1,439/56.8% | 22,686/53.7% | 1.17 (1.05, 1.31) |
| missing | 4 | 1 |  |
|  |  |  |  |
| Low risk (20-34 y/o) | 1,880/74.6% | 34,985/83.7% | Ref. |
| High risk (12-19 y/o, 35-49 y/o) | 650/25.4% | 7,224/16.3% | 1.45 (1.29, 1.63) |
|  |  |  |  |
| Mother attended > 5 years of school | 946/39.7% | 25,114/63.2% | Ref. |
| Mother attended < 5 years of schooling | 1,584/60.3% | 17,091/36.9% | 1.45 (1.29, 1.63) |
| missing | 0 | 4 |  |
|  |  |  |  |
| Richer states | 756/33% | 20,607/52.1% | Ref. |
| Poorer states^b^ | 1,774/67.0% | 21,624/47.9% | 1.23 (1.06, 1.42) |

^a^ Indeterminate postnatal checkups refers to singleton live births in which mothers reported a newborn postnatal checkup within ten days but the location was unspecified. ^b^ Poorer states are EAGA states: Empowered Action Group and Assam encompass Uttar Pradesh, Bihar, Madhya Pradesh, Rajasthan, Orissa, Assam, Jharkhand, Chhattisgarh, Uttarakhand. The remaining states and union territories are classified as richer states.

^c^ Some obstetric complications included seeking treatment for excessive bleeding, prolonged labour, and breech presentation or reported cesarean section/instrument assisted delivery for obstructed labour.

^d^The numbers presented in table are unweighted and the proportions are weighted.
